# Micro-Level Adaptation, Macro-Level Selection, and the Dynamics of Market Partitioning

**DOI:** 10.1371/journal.pone.0144574

**Published:** 2015-12-14

**Authors:** César García-Díaz, Arjen van Witteloostuijn, Gábor Péli

**Affiliations:** 1 Department of Industrial Engineering, Universidad de los Andes, Bogotá, Colombia; 2 Tilburg School of Economics and Management, Tilburg University, Tilburg, the Netherlands; 3 Faculty of Applied Economics, University of Antwerp, Antwerp, Belgium; 4 Antwerp Management School, University of Antwerp, Antwerp, Belgium; 5 Cardiff Business School, Cardiff University, Cardiff, United Kingdom; 6 Utrecht School of Economics, Utrecht University, Utrecht, the Netherlands; East China University of Science and Technology, CHINA

## Abstract

This paper provides a micro-foundation for dual market structure formation through partitioning processes in marketplaces by developing a computational model of interacting economic agents. We propose an agent-based modeling approach, where firms are adaptive and profit-seeking agents entering into and exiting from the market according to their (lack of) profitability. Our firms are characterized by large and small sunk costs, respectively. They locate their offerings along a unimodal demand distribution over a one-dimensional product variety, with the distribution peak constituting the center and the tails standing for the peripheries. We found that large firms may first advance toward the most abundant demand spot, the market center, and release peripheral positions as predicted by extant dual market explanations. However, we also observed that large firms may then move back toward the market fringes to reduce competitive niche overlap in the center, triggering nonlinear resource occupation behavior. Novel results indicate that resource release dynamics depend on firm-level adaptive capabilities, and that a minimum scale of production for low sunk cost firms is key to the formation of the dual structure.

## Introduction

Many industries feature dual market structures, with a few large companies dominating the market’s center and many smaller enterprises surviving in the market’s periphery. Such dual market structures are associated with high concentration and high firm density (i.e., number of firms). In industrial organization and organization theory, the question as to how dual market structures of two dominant firm types evolve has been studied since a long time [1, 2, 3]. However, to date, alternative explanations circulate in the literature that have not yet been integrated [4], implying that the evolution of dual market structures still are not fully understood [5]. In this paper, we develop an agent-based simulation model to explore different dual market structure explanations, revealing how they can or cannot be integrated, and what additional mechanisms may well play a role.

Hitherto, little has been done in order to fully incorporate dynamic firm behavior in the selection-adaptation interplay in the context of population-level market positioning processes. A comprehensive understanding of the emergence and evolution of specific market structures should include insights from different perspectives. Key is that a microeconomic approach can provide the building blocks for a theory that offers a micro-foundation for macro-level market structuration processes [5, 6, 7]. We argue that the development of such a micro-foundation in the form of explicitly modeling firm-level rules of behavior and interaction may indeed be an important contribution by integrating different arguments in the context of the study of market structures. We seek to integrate firm-level decision-making rules [8] in the context of horizontal product differentiation [9] into an industry-level approach through an agent-based computational model [10,11,12].

Our agent-based simulation model connects micro- and macro-level aspects of dual market formation. It runs in a one-dimensional commodity space [13] with a unimodal (peaked) demand distribution. Firms address audience tastes concerning product variants represented as ordered positions along the axis. We consider entry, competition, and (potential) coexistence of two types of agents: *L*-firms with large sunk costs and *S*-firms with low sunk costs. These agents can also differ in size and in their breadth of offerings for their respective audience (niche width).

This choice of settings relates our model to two extant dual market theories, one from Economics and one from Sociology. The unimodal demand distribution, with the demand peak representing a market center surrounded by peripheries, connects our approach to the (original version) of resource partitioning theory of sociological Organizational Ecology (OE) proposed by [2]. This model version is based on demand release at the market fringes inviting small firm entry to highly concentrated markets.

Another stream of resource partitioning arguments explains how small firms make foothold at the market fringes with the establishment of oppositional identities against large center incumbents [3, 14]. These identities oftentimes include anti-mass production sentiments, like in case of the American microbrewery movement [15]. Please note that from now on, when speaking about resource partitioning, we solely focus on the mechanism based on demand release because of its inherent link to standard economics’ thinking. Letting firms with low/high sunk cost operate in the demand landscape establishes a link to the economic dual structure explanation of [1] in Industrial Organization (IO). By choosing this configuration, we also aim at getting new insights on the commonalities between these theories’ underlying disciplinary domains–IO and OE, respectively–known to exist but unexplored for about two decades [6].

From an IO perspective, [1] explains how game-theoretic equilibria might lead firms to incur short-run (so-called endogenous) sunk costs. This is the result of firms’ profit-maximizing decisions whether or not to invest in advertising or innovation. These costs can be recovered by focusing on brand recognition and increased consumers’ willingness to pay through offering products that are tailored to them (horizontal product differentiation). The equilibrium outcome may be a dual market structure in which two types of firms (or strategies) co-exist. On the one hand, in order to recover the sunk cost investments, high investment firms target high demand areas with the intention of reaping scope economies [4]. Thus, large generalists that address a broad range of customer tastes take over the market’s central region by their investment-intensive offerings.

But firms that cannot afford such huge investments in advertising or R&D play a different game, opting for a radically different strategy. Since product differentiation and brand recognition are not attainable for low-investment firms, these firms at the market’s fringes focus on becoming single-product specialists that operate low-cost strategies. The co-existence of large high-differentiation multi-product generalists along with small low-cost single-product specialists is the essential feature of Sutton’s dual market structure [1].

Alternatively, the resource-partitioning theory of OE explains the emergence of dual structure with narrow-niche (specialist) organizations and broad-niche (generalist) organizations in times of increasing market concentration [2]. The argument is based on three assumptions: (i) consumer demand has a center-periphery distribution; (ii) taste heterogeneity among consumers is sufficiently large; and (iii) the industry exhibits strong scale economies in the center of the market, and strong scope economies across the market center and periphery. This usually means a unimodal demand distribution along taste positions. Note however, that unimodality is not a necessary condition for the emergence of resource partitioning. For example, [16] found a dual-center partitioning in the post-1990 structure of the Bulgarian newspaper industry.

Large-scale firms (oftentimes ‘generalists’ targeting a broad range of consumer tastes) will mostly make use of scale economies and compete for the market center abundant in demand. The increased competition in the center leads to consolidation, i.e., to the forcing out many large players. The survivors can then position closer to the now much less crowded market center, so freeing up positions at the periphery. The bottom line is that the consolidation of large generalists in the center, which increases market concentration, also creates the conditions for specialist proliferation at the market fringes [17, 18].

**Fig 1:**
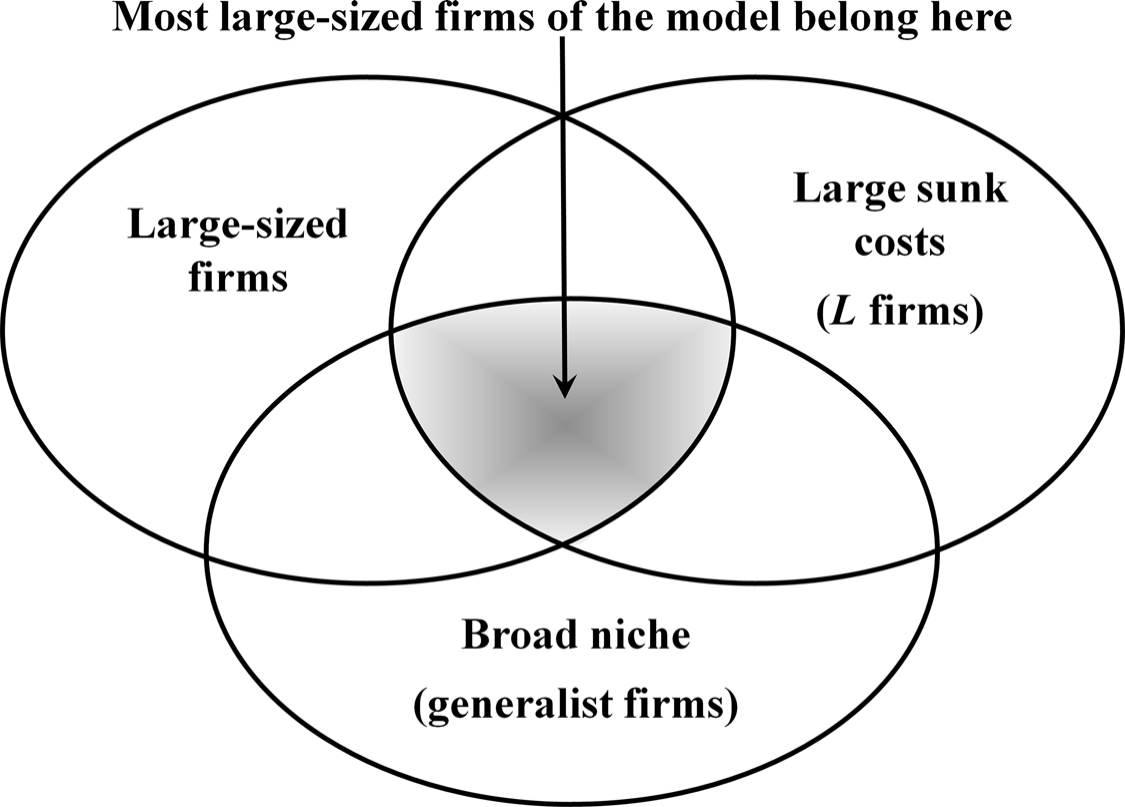
Correspondence between the three firm typologies. As time passes surviving *L* firms tend to be large and have a broad niche, while surviving *S* firms tend to be small and have a narrow niche.

Our simulation model is not, and does not aim to be, a specific computational implementation of these two theories. But in the course of the simulation process, we found remarkably strong correlations evolving with time between their key concepts along a substantially broad range of parameterizations. Our simulations revealed a strong tendency that being large in scale, in scope (niche width) *and* in sunk cost coincide to a large extent (Fig 1), provided that the microeconomic conditions of firms’ entry, exit, offering and competitive engagement assumed in our model apply. Similarly, with the same conditions in place, being small, being specialist and having low sunk cost strongly correlate, too.

This endogenously evolving convergence between our model concepts, as visualized in Fig 1, offers possibilities for exploring linkages between different dual market explanations. We evaluate these commonalities in the concluding part. There, we also discuss explanations for those simulation findings that go beyond extant market partitioning predictions, so broadening the known repertoire of cases/causes of dual market formation. As is the case with all simulation studies [19], the findings are only justified for the given model settings. But within our setting, the reported results are robust: they have been observed under a broad range of parameterizations. Next, we explain the computational model, present the key results, and discuss the conclusions derived from this work.

## The Model

Our model contributes to recent works in computational approaches to markets, which include examples of industry evolution [10], agent-based product diffusion [20], and market failures [21], among others. To prevent being overloaded with technicalities, the main text describes the essence of the model and highlights how the corresponding formal constructs work. The detailed formal account of the model’s equations, parameter descriptions and their value ranges applied at sensitivity analyses is available in the S1 File. The model consists of a series of files written in Matlab, which are available upon request.

Close to the spirit of evolutionary games, we study the evolution of performance of two market strategies differentiated by initial sunk cost investment. Firms compete in a market characterized by scale economies and niche-width (scope) diseconomies. We take the work of [4] as our steppingstone, who argue that the large / small sunk cost firm types reproduce dual market structures in multi-product settings, similar to the generalist / specialist context in resource partitioning. So, linking to the IO literature [1], we consider large versus small sunk cost types: large sunk cost firms can and small sunk cost firms cannot benefit from scale economies. Specifically, firms with large sunk costs (*L* firms) invest in large production capacity and aim to be efficient in the long run. Firms with small sunk costs (*S* firms) are cost-efficient at the time of market entry, but their sunk cost investment is insufficient to be cost-efficient in the long run. Due to the niche-width related negative effects, as we will explain below, *S* firms take advantage of a strategic location at the market fringe, where scale-based competitors have no efficient reach because the scope diseconomies of niche spanning cannot be compensated by scale economies. An additional reason to keep only two market strategies comes from knowing that significant firm entry diversity, represented by very few and contrasting firm types, is needed to generate dual market structures with few dominant firms at the market center and a considerable number of small players at the periphery (cf. [22]).

Firms offer a single price for their whole niche. Scale advantages are translated into lower prices, and consumers buy from the cheapest producer in their niche. Firms use a markup price in order to reflect their scale advantages, provided that such a price does not exceed the consumer’s participation constraint. Consumers also take into account the negative effect of product dissimilarity, which is the distance between the firm’s niche center and the consumer’s location. Firms seek to increase their scale advantage by expanding their niches, but large-niche firms need to offer low enough prices in order to keep consumers at the niche edges satisfied.

### Attribute Space and Location Specification at Entry

Some attribute space models consider organizations’ clientele distributed across variables in an *N*-dimensional *Blau-space* of socio-demographic characteristics [23]. Our space representation is not sensitive to the choice as to whether the demand curve is drawn over socio-demographic characteristics or taste positions. Of course, spatial representations have different connotations in the literature, depending on the context. Other used renderings may be as straightforward as a geographical space [24], or as abstract as an NK landscape [25,26]. Our attribute space is a commodity space [13] with one product dimension along which each firm offers a single product or service. Customers’ taste preferences are represented by their ideal points along this single dimension. The simulation model always starts with one single firm, and firms enter the market at a constant rate *x* per time period if the space is not completely occupied. The entry rate varies from 2 to 3. Identifying a simulation step with one month time, this range setting fits, for example, the average entry rates in the American automobile industry, which has exhibited dual market characteristics over its history [3]. The American automobile industry registered approximately two thousand active firms over its first hundred years of existence [27]. The attribute space corresponds to a unimodal distribution of consumers with *b*_*k*_, *k* = 1, 2, …, *N*, denoting the magnitude of demand, and *N* representing the total number of taste positions.

The attribute space is furnished with demand according to a beta distribution with parameters *α’* = *β’* = *η > 0*. This assures a unimodal distribution with a finite number of taste positions. For the baseline model, we take *η =* 3 and *N* = 100. All simulation runs are performed using total demand of *∑*_*k*_ *b*_*k*_ = 5,500 consumers. Firms that enter the market set their product niche location (niche center) according to the probability distribution of non-served consumers: higher crowding at a taste location tends to repel entrants.

### Firm’s Cost Structure

Building/securing market positions involves costs that increase with the breadth of the niche the firm establishes or sustains. Accordingly, our single-product firms have two-piece cost functions. One component reflects the production costs *C* ^*i*^_*PROD*,*t*_, while the other accounts for niche-width costs, *C* ^*i*^_*NW*,*t*_:

$$C_{t}^{i}=C_{PROD,t}^{i}+C_{NW,t}^{i}.$$ (1)

Production level of firm *i* at time *t* (*Q*_*it*_) is given by a Cobb-Douglas function:

$$Q_{i,t}=F_{i}^{\alpha }V_{i,t}{}^{\beta }.$$ (2)

*F* and *V* denote production factor quantities that contribute to fixed (sunk) and variable costs, respectively. Firms derive their production costs (*C* ^*i*^_*PROD*,*t*_) from the long-run average cost curve (*LRAC*) of the whole industry. The *LRAC* curve is the envelope of the most efficient production possibilities in the industry. We assume that *α* + *β* > 1 in order to have a downward-sloping *LRAC* and so positive scale economies. Production costs for the firm are calculated according to the usage of production factors *F* and *V* that the firm needs to produce quantity *Q*. That is, assuming that production factor prices are *W*_*F*_ and *W*_*V*_, respectively, the *LRAC* curve is calculated by solving the following optimization problem:

$$\begin{array}{cc} \min & W_{F}F_{i}+W_{V}V_{i}\\ s.t. & Q_{i,t}=F_{i}^{\alpha }V_{i,t}{}^{\beta } \end{array}.$$ (3)

The production cost of every firm *i*, *C* ^*i*^_*PROD*,*t*_, is computed assuming that the firm has a fixed usage of factor *F*, independently from production levels. Therefore, *W*_*F*_ *F*_*i*_ stands for the fixed costs of *i*). We consider two discrete levels of *F*, large and small, that firms can take with equal probability. These levels define the two types of agents in the model: *L* firms and *S* firms are characterized by high and low levels of fixed cost-related production factors, respectively. Niche-width costs represent the negative effect of producing for a broad range of consumer preferences. For instance, attempting to serve a greater variety of tastes may involve additional advertising and merchandising costs. Thus the cost of serving a consumer taste portfolio increases with taste heterogeneity. Firms’ niche-width costs are proportional to their niche span:

$$C_{NW,t}^{i}=NWC\left\|w_{i,t}^{u}-w_{i,t}^{l}\right\|,$$ (4)

where *NWC* is constant, ‖.‖ denotes Euclidean distance. In the present one-dimensional space, *w* ^*l*^_*i*,*t*_ and *w* ^*u*^_*i*,*t*_ represent, respectively, the lower and upper niche limits of firm *i* at *t*. A firm’s niche center stands halfway between the lower and upper niche limits.

### Consumer Behavior

Each consumer buys once every time period. *S*_*k*,*t*_ denotes the set of firms with an offering at position *k* at *t*. Just like in Hotelling-type address models [9, 13], consumers’ displease increases with the distance between their ideal taste point and the offering. Their ‘product dissimilarity costs’ are measured as the distance between their ideal point and the product location. Accordingly, consumers buy from the firm that offers the lowest U*_k,*t*_ compound cost (price plus product dissimilarity):

$$U_{k,t}^{*}=\begin{array}{c} \min\\ i\in S_{k,t} \end{array}\left\{P_{t}^{i}+\gamma \frac{\left\|nc_{t}^{i}-k\right\|}{\left(N-1\right)}\right\},$$ (5)

where *P* ^*i*^_*t*_ is firm *i*’s price at time *t*. Product dissimilarity is normalized by the maximum possible Euclidean distance in the product space, *N–* 1.

If the selected firm cannot fully satisfy demand, the consumer buys from the second best alternative, and so on. The model does not allow that consumers abstain from purchasing, so spending their money on an ‘outside good’ [28]. Since the *LRAC* curve reflects the efficient production frontier (in terms of costs) as a function of quantity, and the curve is downward-sloping as *Q* increases, the highest cost value is the one that corresponds to the smallest quantity. Therefore, we assume that this maximum cost value–the smallest efficient production quantity–is a reference point of the maximum price a consumer is willing to pay: *P*_*max*_ = (1+*φ*)*LRAC*│_Q = 1_. In other words, consumers will not pay a price beyond the highest unit cost (reflected by the *LRAC* curve) multiplied with 1 + *φ*, where coefficient *φ* stands for mark-up pricing [29]. The value of *φ* is set between 0 and 1. We use *φ* = 0.2 for our baseline model. If a consumer buys from firm i*, the price *P* ^i*^_t_ paid cannot exceed this maximum price compensated by the negative effect of product distance from the customer’s ideal taste point:

$$P_{t}^{i*}\leq P_{\max}-\gamma \frac{\left\|nc_{t}^{i}-k\right\|}{\left(N-1\right)}.$$ (6)

In order to reflect scale advantages, firms use a mark-up price over average costs given as (1+*φ*)*C*(*Q*)/*Q*, with *C*(*Q*) denoting the firm’s total costs when producing quantity *Q*. Therefore, a firm will set a price *P* ^i*^_t_ that is the lowest of the following two: its markup price and the maximum bearable price the most distant consumer within its niche would pay (Eq (7)):

$$P_{t}^{i*}=\min\left[P_{\max}-\gamma \frac{\left\|nc_{t}^{i}-k\right\|}{\left(N-1\right)},(1+\phi )C(Q)/Q\right]$$ (7)

### Entry Price Setup

Firms enter at one single position in space, searching for a competitor-free foothold to enter the market. Thus, entrants look for residual demand (non-served consumers) in space. Entry probability at a location increases with the size of residual demand. Knowing the percentage of demand of each potential entry position *k* that has been already served, *CBP*_*k*_, the firm calculates its potential production quantity based on the amount of residual demand at *k*, *Q* = (1—*CBP*_*k*_)*b*_*k*_. Subsequently, the firm sets the unit price as described in Eq (7) above. Since *S* firms have lower fixed costs to cover, they are able to make profits at the time they enter the market. In contrast, *L* firms may need some time to reach an operation scale that allows them to generate positive profit. Thus, we assume that *L* firms have some initial endowment that helps them going through this growth period [30]. The magnitude of their endowment *E* is measured by the number of time units during which a firm can survive without sales. For the baseline model, we set *E* = 12.

### Firm Expansion

Firms can expand horizontally (in breadth) and vertically (in depth). Horizontal expansion takes place through widening the niche, while vertical expansion means increasing sales within the given niche. In line with behavioral theories of bounded rationality [31, 32], we assume a simple search heuristic with which firms can circumvent complex optimization tasks. Our firms are prudent observers that base their actions on their rivals’ past behavior [33]. The expansion being either horizontal or vertical, the firm first decides upon a target quantity based on the latest observed prices and costs. Subsequently, the firm computes expected incremental profits and decides if expansion is likely to have positive returns.

As said, vertical expansion is a production quantity adjustment at fixed niche breadth. At time *t*, firm *i* makes production adjustments for the next round *t*+1 targeting the residual demand Δ*Q*_*v*,*t+1*_ in its current niche. Then, it evaluates whether incremental revenues surpass incremental costs. If so, the firm decides to expand. Another modeling possibility would be that firms also target absorbed demand (i.e., demand already served by other firms). However, this would involve modeling strategic pricing behavior, which would dramatically complicate our model.

Horizontal expansion takes place by increasing niche width. The firm estimates target quantities Δ*Q* ^*u*^_*h*,*t+1*_ and Δ*Q* ^*l*^_*h*,*t+1*_ at both side of its current niche, and decides to expand in the more attractive direction–if there is any. For convenience, we assume that niches have no discontinuities, so specifying the upper and the lower niche limits is enough to characterize the set of positions the firm serves.

Niche expansion is controlled by expansion probability, *ExpCoef*. Since we first run the model for 2,000 time periods, our criterion is that an *L* firm should be allowed to have enough time to fully expand up to its “fundamental niche” (i.e., the niche the firm would cover in absence of competition), even if it enters the market at a mature state (> 1,000 time periods). Otherwise we might not observe a population of growing *L* firms in the model. Values for the expansion coefficient were chosen between 0.03 and 0.05.

The quantities Δ*Q* ^*u*^_*h*,*t+1*_ and Δ*Q* ^*l*^_*h*,*t+1*_ are set as follows. Consider the following example: assume that there are two firms, *A* and *B*, serving the same taste position. The position has a total demand of ten consumers, and the compound costs consumers perceive at that position are *U*(*A*) = 10 and *U*(*B*) = 15 with captured demands *Q*(*A*) = 7 and *Q*(*B*) = 3. If firm *C* attempts to enter that position, and assuming that the combination of price and dissimilarity result in offering a cost of *U*(*C*) = 12 at that position, the ascendant cost ranking will place firms in the following order: *A*, *C*, and *B*. We assume that the firm estimates its quantities assuming that offered quantities and costs of rivals remain the same as the last round. Thus, firm *C* estimates that *A* will keep its previous period’s demand in the next round (i.e., *Q*(*A*) = 7), since *A* still has the cheapest offer. But now, given that *C* has a better offer than *B*, *C* would steal B’s demand and estimate that in the next round *Q*´(*C*) = 3 and *Q*´(*B*) = 0. Therefore, firm *C* estimates a quantity of 3 at that taste position. It is important to note that estimations might not match realizations, so a firm might produce a quantity for a position that might not be able to sell later. Also, since transactions are executed across positions in a random order, a firm may sell all its produced quantities at other taste positions, possibly leaving for a given taste position no quantities to sell, even if the offered cost at that position is the lowest. In a similar fashion throughout the simulation, firms estimate their next round’s demand for the case of horizontal expansion, and quantify its benefits by calculating potential incremental profits.

Since firms may gain positions due to expansion, they occasionally re-compute their niche centers as new positions are added. In addition, firms may lose positions through competition: a position at the upper or lower niche border is considered lost when the firm’s realized demand is zero there. In summary, the niche center can be relocated as firms gain or lose niche positions. The newly offered price accounts for the new distance compensation for customers at their niche location.

Niche expansion and endowment provide different adaptive capacities to the firm. In order to illustrate their respective effects, we have run a number of simulations with only one large sunk cost firm. Below we present results from two scenarios: (i) one with the baseline endowment value (12) by which we compared the implications of low expansion capabilities (0.03) vis-à-vis high expansion capabilities (0.05); and (ii) another one with low expansion capabilities (0.03), by which we compared the impacts of low (6) and high (18) endowment levels. Each scenario has been tested in 60 simulations.

**Fig 2:**
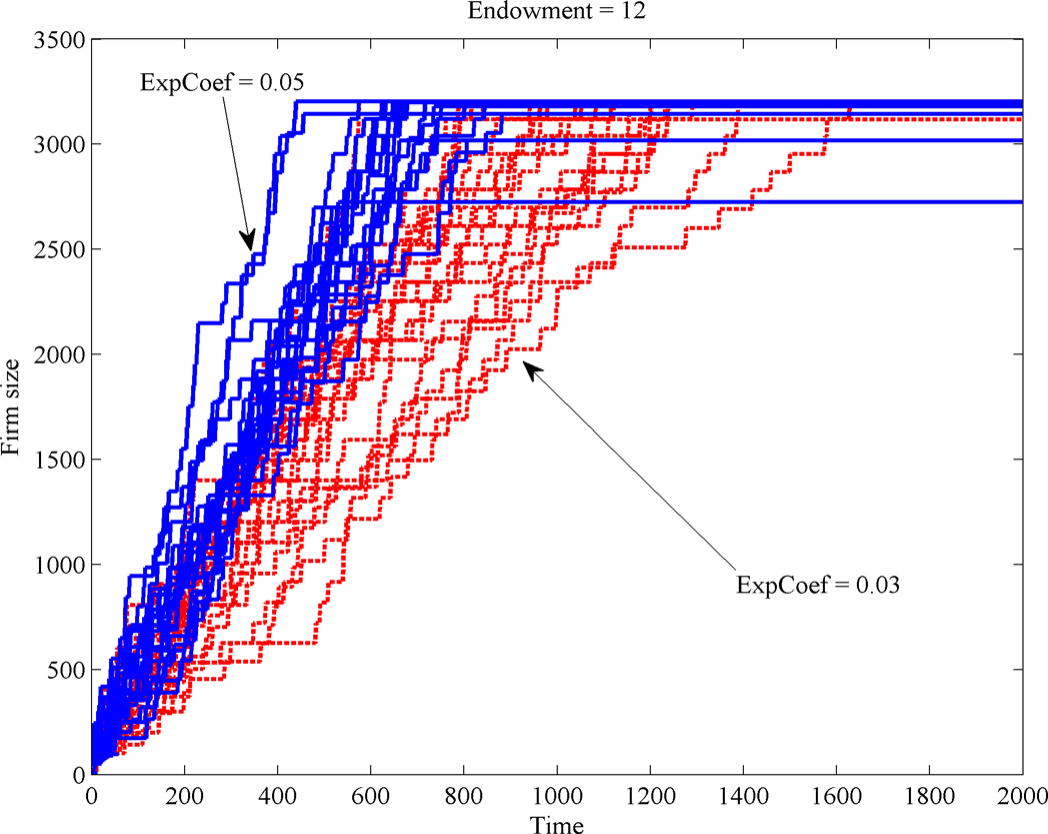
Firm size change with low and high expansion coefficients.

If we consider sold quantities as the indicator of firm size, different expansion coefficients imply different growth speeds. The first scenario (i), which is depicted in Fig 2, illustrates how firms grow with different expansion coefficients in place.

**Fig 3:**
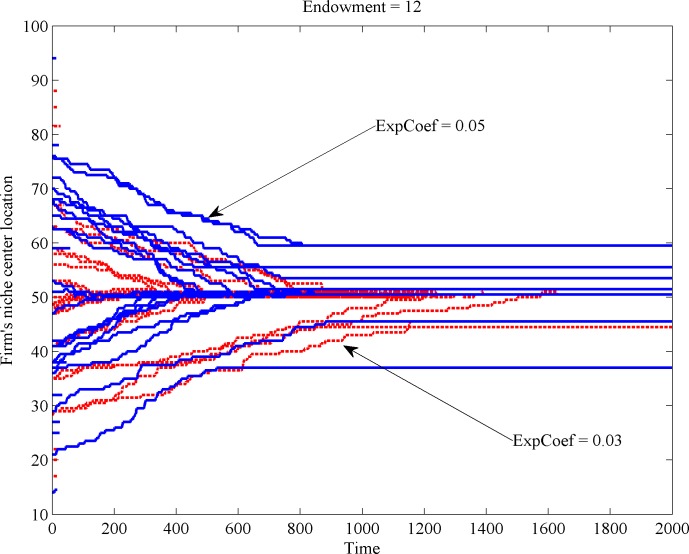
Firm’s niche center change with low and high expansion coefficients.

The observed difference in growth behavior has implications as to the expected location of the firm in the long run. Fig 3 illustrates the relocation of firm’s niche center over time (recall that the space has a unimodal shape and comprises 100 different taste positions, numbered from 1 to 100). Fig 3 reveals that firms with low expansion coefficients (dotted lines) only reach the market center (located at position 50) if their initial position is not too distant from the center. Firms with a large expansion coefficient (continuous, blue lines) manage to reach a near center positions even from distant spots of scarcer resource.

**Fig 4:**
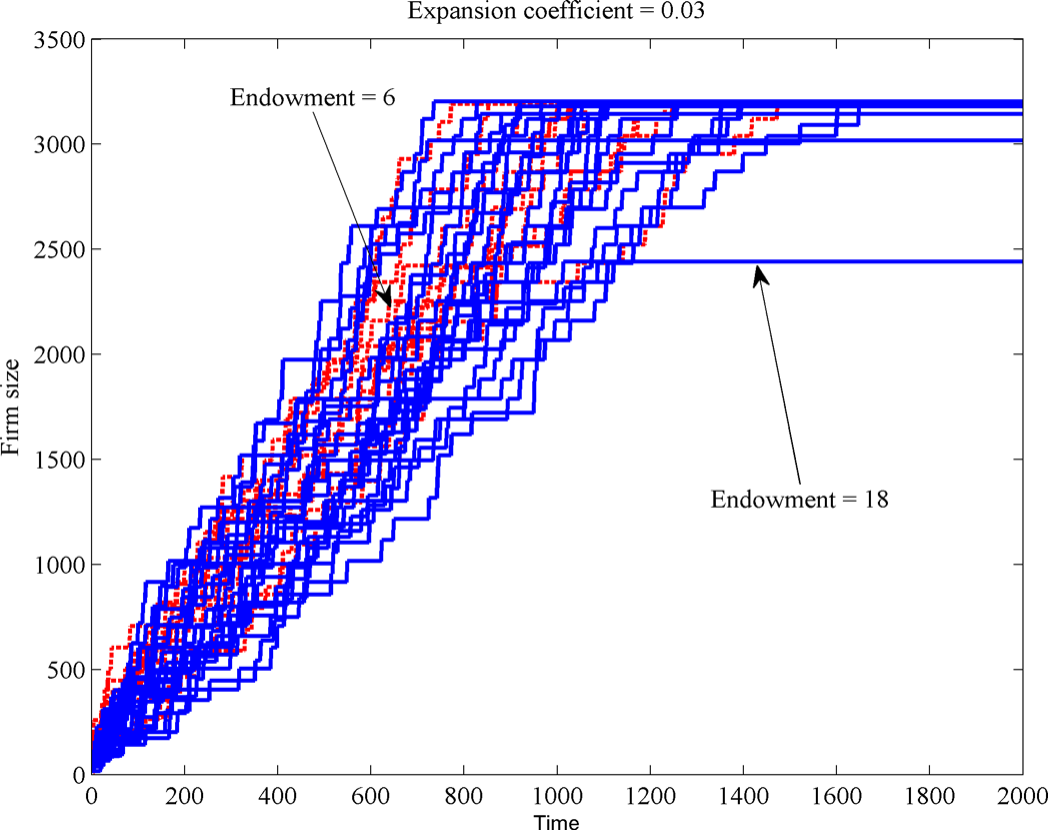
The effect of different endowment levels on firm size.

**Fig 5:**
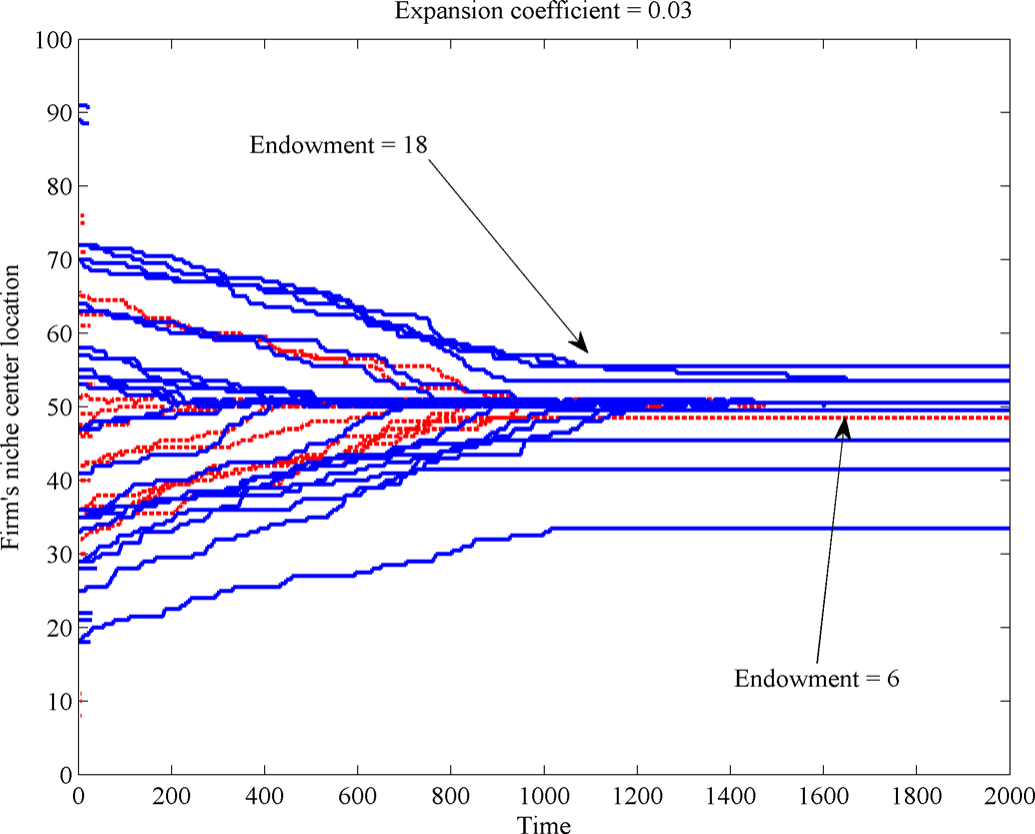
The effect of different endowment levels on niche center location.

Let us consider the second scenario. Figs 4 and 5 illustrate the effect of different endowment levels. For the sake of discriminating the effects of low and high endowment, we chose for fixed, low expansion coefficient (0.03). Fig 4 illustrates that even with equal expansion capabilities in place, the future firm location might depend on the endowment level. Firms with low endowment (dotted lines) survive the whole 2000 steps simulation horizon in only 37% of the cases, while firms with large endowment (continuous lines) survive in 80% of the cases. This is why Figs 4 and 5 appear to show more continuous than dotted lines: many lowly-endowed firms do not reach the end of the simulation.

Fig 5 indicates that firms with low endowment that are born in semi-center positions manage to survive and move toward the center. Firms with low endowment that are born far from the center eventually die in about 63% of the cases. Firms with higher endowment born farther from the center can still manage to reach the near center. Only 20% of the highly-endowed firms die during the whole simulation period.

## Experimental Design

We perform a hazard rate and a regression analysis of parameter variations on our key model variables. The hazard rate analysis is carried out to inspect the determinants of firm mortality. We specify an event-history model, in which the dependent variable is the instantaneous exit rate at time *t*, provided that firm is alive at *t*:

$$h\left(t\right)=\lim_{dt\to \infty }\frac{P\left(t\leq exit\leq t+dt\right)|exit\geq t)}{dt}$$ (8)

Following the traditional procedure in ecological studies [34], we estimate a piecewise constant exponential hazard rate model where each estimated constant matches a given duration interval of the firm lifetime. In order to calculate the number of intervals, we take into account that firms may have a strongly age-sensitive baseline hazard function when they are very young, and subsequent longer spells with a more stable hazard as they grow older. Therefore, we define a fine-grained youth period characterized by short time intervals, along with longer intervals for older ages: [0,10), [10,20), [20,30), [30,40), [40,50), [50,60), [60,70), [70,80), [80,90), [90,100), [100,200) and [200,∞). We estimate baseline mortality numbers represented by a set of constants {*ϕ*_1,1_, *ϕ*_1,2_, *ϕ*_1,3_,…}, each of them corresponding to an interval *l* (*l* = 1, 2,…), plus a column vector *ϕ*_2_ that is associated with the row vector of covariates Z. Mortality at interval *l* is accounted by:

$$h\left(t\right)=\exp(\phi_{1,l}+Z\phi_{2})$$ (9)

Our covariates are niche width (*NW*), firm size (*Size*, measured as sold volume), distance to market center (*DC*), market concentration (*Gini*) and firm type (*Type* = 0 for *L* firms, and *Type* = 1 for *S* firms). It is important to note that, to increase robustness, we have experimented with both the C_4_ ratio and the Gini coefficient as concentration measures. Oftentimes, we found very high correlation between the two; see the visual results in Section 5. However, since the Gini coefficient reveals a clear monotonic behavior, we preferred to use this as a proxy for market concentration in the statistical analyses. As control variables, we include industry age (*Indage*), active market size (*Mass*, measured as total sold volume), and firm density (the active population of firms in the market). After inspection of correlation matrices across all simulations, we decided to remove niche width from the list of variables as this revealed a high positive correlation with firm size. So, as indicated above, in our model, broad niche firms (generalists) are large, and small firms have narrow niche (specialists). We also found a strong positive correlation between firm density and market concentration. Consequently, firm density has been eliminated from the list of control variables, too.

First, we study the hazard rate effects in different representative scenarios defined according to variations in parameters such as small sunk cost investment (*Q*_*S*_), product dissimilarity (*γ*), endowment (*E*), entry rate (*X*), markup value (*φ*), and expansion probability (*ExpCoef*). It is worth mentioning that in this analysis we do not use an experimental design where responses of all possible parameter value combinations are aggregated into a sort of a pooled estimation. Each parameter combination generates different market concentration effects on the mortality of both firm types and the interplay between these mortality levels. For that reason we rather opt for working with a number of scenarios that represent different particular instances of dual market emergence. Given the number of parameters of interest, we varied each parameter at a time departing from a baseline scenario (Scenario 1). We thus obtain a total of 13 scenarios of interest. In summary, these scenarios correspond to models with mid-range parameters or baseline values (Scenario 1), and to models with variations in small sunk cost investment (Scenarios 2, 3 and 4), markup (Scenarios 5 and 6), product dissimilarity (Scenarios 7 and 8), endowment (Scenarios 9 and 10), probability of expansion (Scenarios 11 and 12) and entry rate (Scenario 13). Detailed parameter specifications for all scenarios are provided in Table 1. Additionally, we examine the behavior of our main time-evolving variables of interest–i.e., market concentration, per-type density, and per-type total covered market space. Each scenario has been run 30 times for 2,000 time periods. Each run produced more than 100,000 duration-related observations.

**Table 1:** Parameter values for hazard rate analysis.

Parameter	Definition	1	2	3	4	5	6	7	8	9	10	11	12	13
*Q*_*SS*_	Small sunk cost	10	**5**	**15**	**20**	10	10	10	10	10	10	10	10	10
*φ*	Markup	0.2	0.2	0.2	0.2	**0.1**	**0.3**	0.2	0.2	0.2	0.2	0.2	0.2	0.2
*γ*	Product dissimilarity	60	60	60	60	60	60	**50**	**70**	60	60	60	60	60
*E*	Endowment	12	12	12	12	12	12	12	12	**6**	**18**	12	12	12
*ExpCoef*	Expansion probability	0.04	0.04	0.04	0.04	0.04	0.04	0.04	0.04	0.04	0.04	**0.03**	**0.05**	0.04
*x*	Entry rate	3	3	3	3	3	3	3	3	3	3	3	3	**2**

We measure the occupied space per firm type as the aggregated number of niche positions in which this firm type realizes sales. When *L* firms extend their niche towards central positions with the many consumers, they abandon taste positions with scarce demand at the edges, provided that the benefits of utilizing this demand would not counterbalance the increased scope costs caused by their extension. Accordingly, we measure the space released for *S* firms by the contraction of total space occupied by *L* firms.

The regression analysis aims at looking patterns when concentration has stabilize and considers (i) market concentration, (ii) *L* firm density, (iii) *S* firm density, and (iv) *L* firm space contraction as dependent variables. The independent variables have been small sunk cost (*Q*_*S*_), product dissimilarity (*γ*), markup (*φ*), endowment (*E*), expansion probability (*ExpCoef*), and entry rate (*X*). The description of the 4 x 3^4^ x 2 = 648 parameter value combinations is in the S1 File. We follow standard simulation procedures to explore model behavior under different parameterizations (for details, see [35]), and estimate OLS regression coefficients *B*_*i*_ with the variables mentioned above. Thus, the regression equations (one for each dependent variable *y*_*i*_, i = 1,…4, listed above) have the form of:

$$y_{i}=A+B_{i,1}Q_{S}+B_{i,2}\gamma +B_{i,3}\varphi +B_{i,4}E+B_{i,5}ExpCoef+B_{i,6}X,\;i=1,\ldots ,4$$ (10)

Every parameter combination is executed five times, giving 3,240 observations in total. To better capture steady-state parameter variation effects, we have run each simulation for 5,000 time periods, averaging the key outcome variables for the last 500 time units.

## Results

This section presents the main results, divided in four blocks. First, we refer to the changes of market concentration and market structure (according to observations of the scenarios defined above); second, we evaluate the effects of scale-based competition at the market center and its impact on resource release; third, we reflect on the hazard rate and regression analysis results. The outcomes reported below are common patterns found in all scenarios.

### Concentration and Market Structure

In line with many ecological empirical studies, our market partitioning approach is not focused on equilibrium but on the dynamic effects of increasing market concentration. Thus our aim was to inspect how firm mortality is affected by rising concentration. Therefore, we took the level of market concentration as the main criterion for stopping the simulation. We observed that Gini and C4 concentration ratios tend to stabilize, at least at an initial phase.

**Fig 6:**
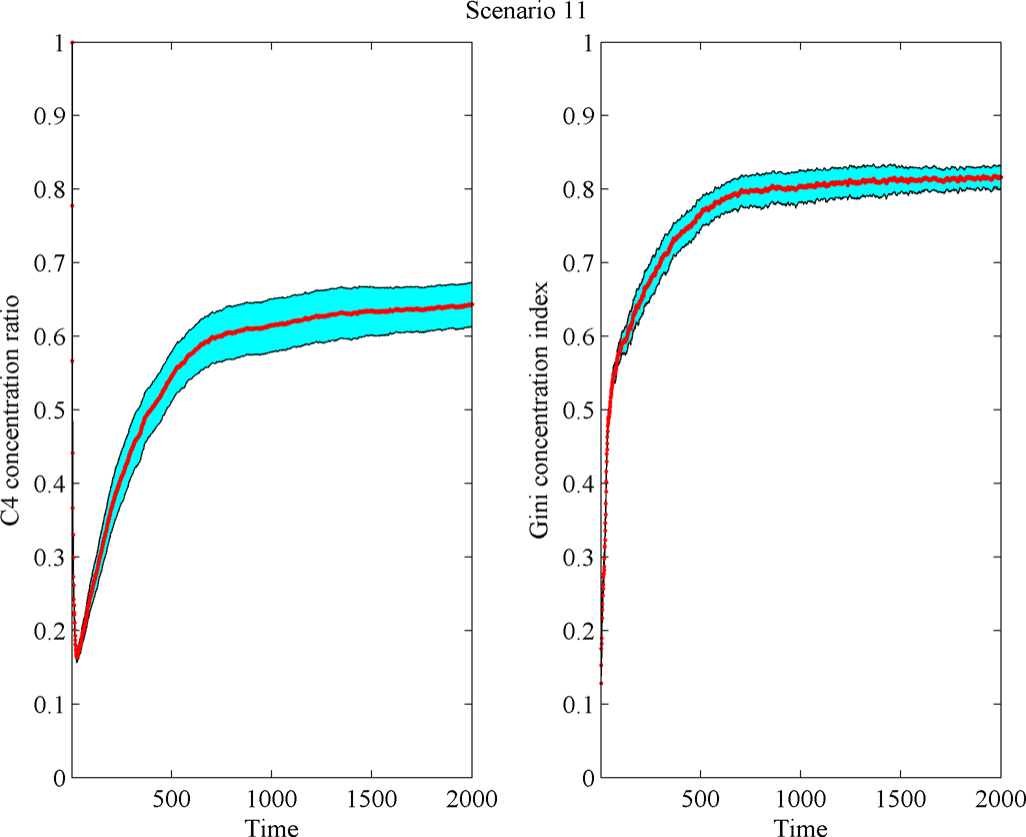
Average model behavior in Scenario 11 (shadowed regions correspond to confidence intervals). Left panel: C4 concentration ratio; right panel: Gini coefficient.

**Fig 7:**
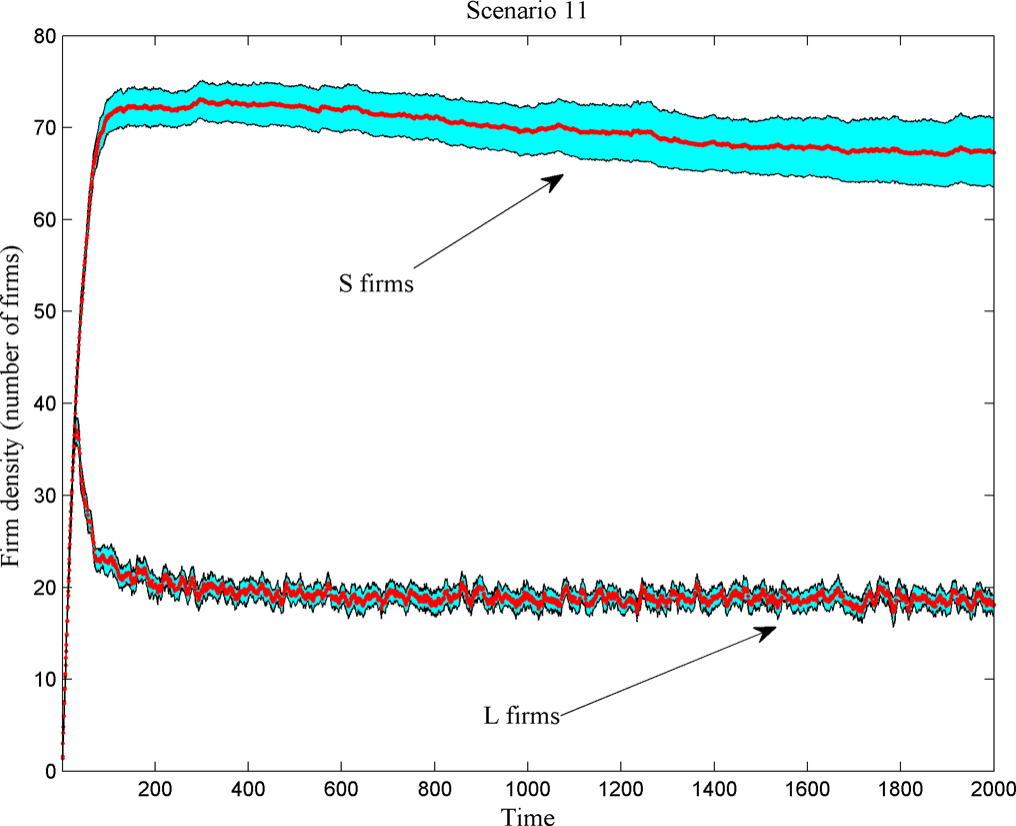
Number of *L* and *S* firms (shadowed regions correspond to confidence intervals).

As said, market concentration was measured with the compound share of the four largest firms (C_4_) and with the Gini coefficient. C_4_ first declines as the market gets populated with firms (recall that the model starts with only one firm), then it turns increasing as dominant *L* firms gain market share and grow large (Fig 6). While *L* firm density declines, *S* firm density first rapidly increases and then slows down gradually. After an initial increase, the pace of concentration growth also slows down, featuring temporary stability. Subsequently, a dual market structure emerges (Fig 6). These evolution patterns prove to be fairly consistent across all scenarios. Fig 7 displays a representative sample of density change patterns. The thick line represents average (mean) behavior. The shadowed regions are confidence intervals at 95% over the mean. All results reported below apply to markets with a unimodal demand distribution and type heterogeneity with *L* and *S* firms.

By way of robustness check, we have run simulations up to 50,000 time steps and have found that convergence has not always been achieved. Concentration may either fully stabilize or begins to oscillate (but even not necessarily around a constant mean). These computational results suggest that dual market structures are not necessarily stable in the very long run: the market might become an oligopoly or might become fragmented.

**Fig 8:**
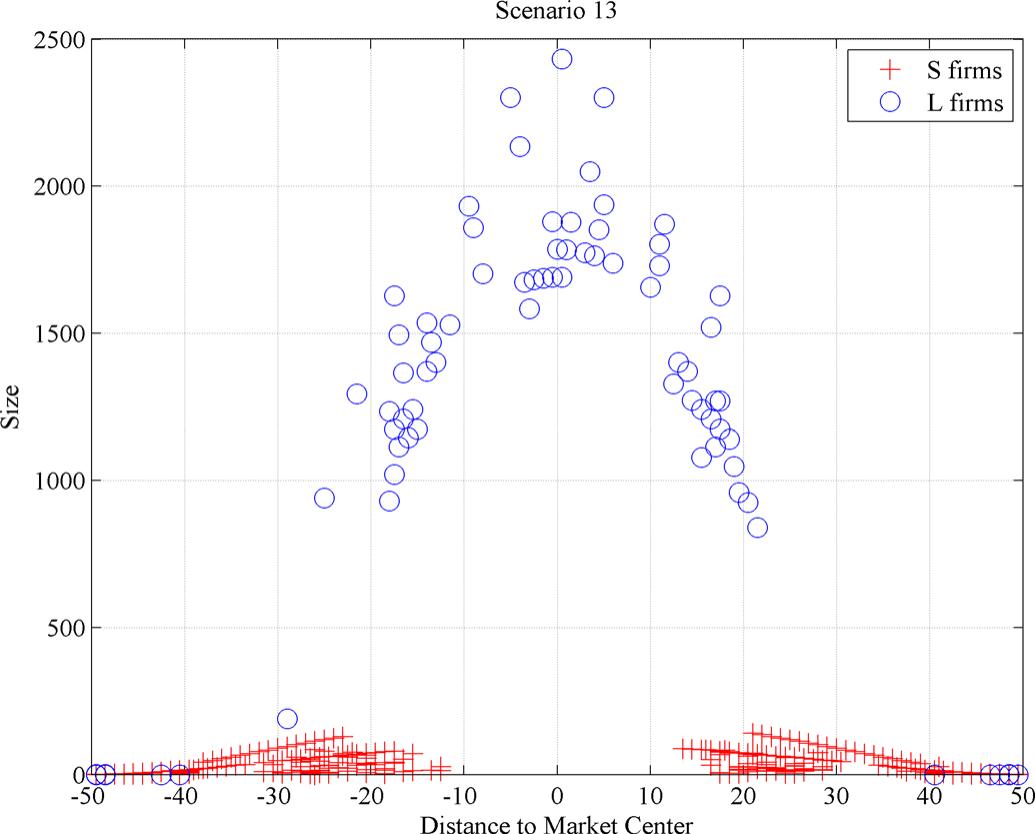
Size distribution per firm type (aggregated data on all simulations for this scenario).

*L* firms’ number reaches a peak before scale-based competition triggers a decrease. The number of *S* firms follows an S-shape curve, first soaring with *L* firm shake-out and then settling afterwards (even turning mildly declining under some scenarios; see Fig 7). Along the simulation period, the numbers sustain for both types, indicating the existence of type-specific carrying capacities for *L*- and *S*-firms. Fig 8 displays the space-related selection effects of scale-based competition. We plot the size distribution of firms vis-à-vis their distance from the market center across different scenarios. Large-sized, broad-niche *L* firms reside in the market center, completely kicking out *S* firms from the center, while narrow-niche players (with a dominant population of *S* firms) proliferate at the market fringes.

#### Outcome 1

(a) As the market gets crowded, market concentration increases; (b) Large sunk cost (*L*) firm density first increases and then declines, while small sunk cost firm (*S*) density increases; (c) broad-niche firms (typically of the *L* type) take over the center, whilst narrow-niche firms (a mixture of *L* and *S* firms) locate at the market fringes, producing a dual market structure, with narrow-niche firms’ density systematically being higher.

### Scale-Based Competition and Space Release

**Fig 9:**
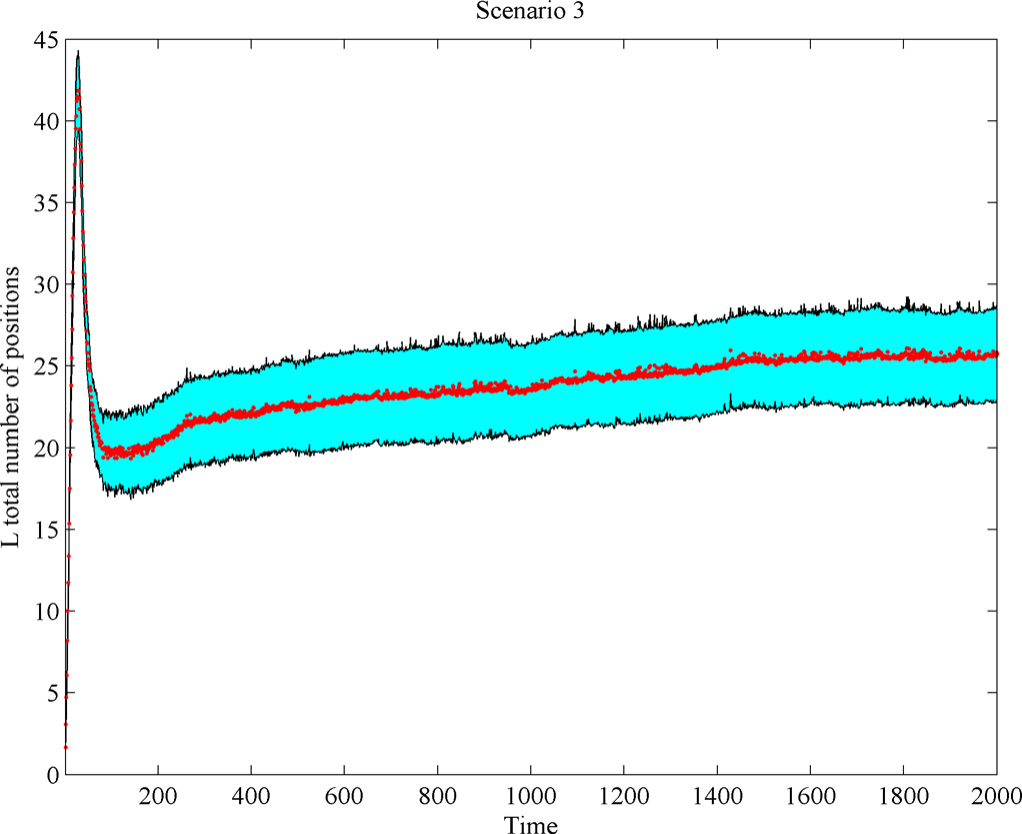
*L*-firm space occupancy over time in Scenario 3 (shadowed regions correspond to confidence intervals).

**Fig 10:**
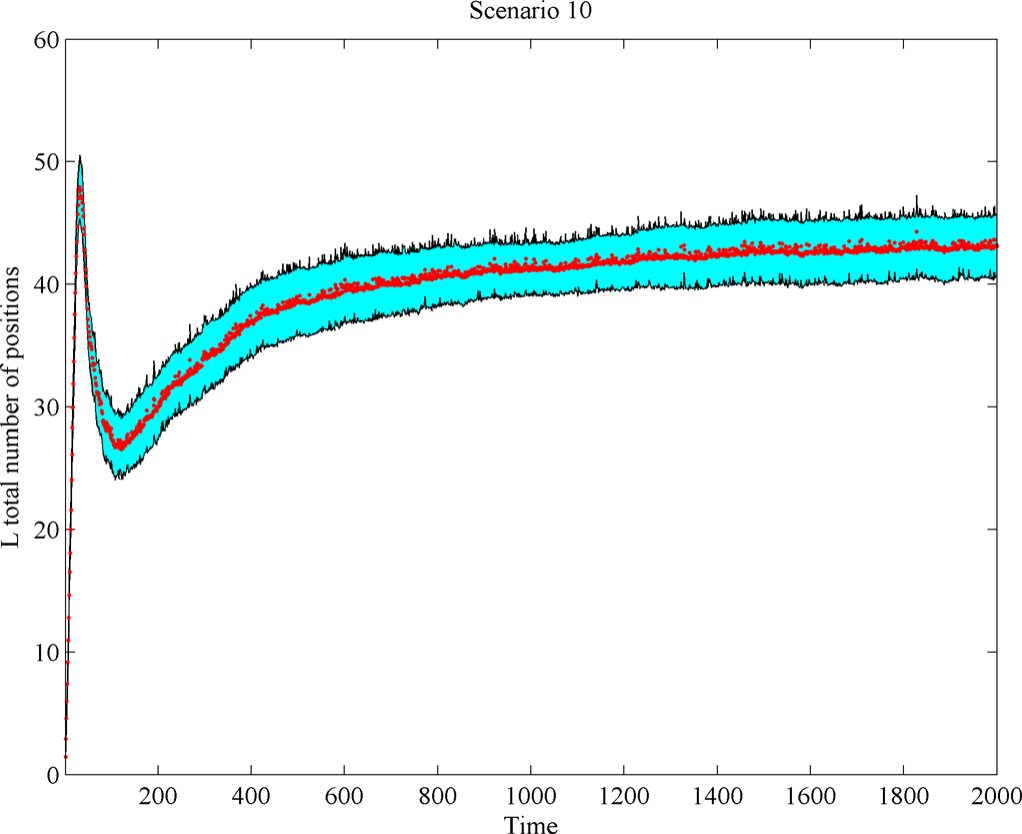
*L*-firm space occupancy over time in Scenario 10 (shadowed regions correspond to confidence intervals).

Next, we analyze the change pattern of *L* firm space coverage over time. We investigate whether *L* firms move toward the center and whether their involvement in scale-competition indeed generates space release at the market’s peripheries. We found that while *L* firm density declines after reaching a peak, the total space occupied by *L* firms first arrives at a maximum, then declines, and finally increases again. The few *L* firm survivors normally keep growing and continue to conquer additional niche positions. As a result, the total *L* firm space volume change oftentimes reveals a non-linear behavior, as can be seen in Figs 9 and 10.

#### Outcome 2

Scale-based competition in the market center may cause space release at the market’s semi-periphery. But in the long run, large firms re-occupy (some of) the abandoned space.

Strong firm’s shake-out at central market positions pulls the surviving large firms toward the center, so igniting demand release at the market fringes. Our results also indicate that a portion of the *L* firms return to the (semi)peripheries. There is an intuitive explanation for this ‘pendulum’ effect. The downfall of large firms frees up a substantial slice of once-served demand, pulling the surviving *L* firms toward the center. Since survivors act similarly, this may lead to a kind of overshoot effect: center competition will increase, again, dramatically, making niche extensions toward the peripheries profitable. Also, surviving firms may have not yet reached their minimum efficient scale at the moment of departure of dying firms: i.e., survivors may still be able to decrease unit production costs by expanding. Such a reduction in unit costs may still dominate over scope diseconomies, allowing survivors to capture more demand than had been released before by departing firms. This dynamics contributes to a loss, and then to a subsequent recovery, of demand by market-center players.

### Hazard Rate Analysis

We performed a hazard rate analysis for each individual simulation. Then, each coefficient was averaged over all simulation runs of the same scenario. Average coefficient values, the standard deviations and the percentages of time for which the variables have been found significant are provided in Table 2.

**Table 2:** Hazard rate analysis coefficients.

		Size			Gini			DC			Type	
Sc.	Coef.	% Sig.	Std.Dev	Coef.	% Sig.	Std.Dev	Coef.	% Sig.	Std.Dev	Coef.	% Sig.	Std.Dev
1	**-0.17**	100%	0.04	**4.52**	100%	2.11	**1.23**	83%	1.02	**3.88**	100%	0.33
2	**-0.20**	100%	0.05	**7.70**	100%	1.53	**0.69**	57%	1.05	**4.35**	100%	0.18
3	**-0.15**	100%	0.03	**5.39**	100%	3.31	**0.62**	70%	0.82	**3.96**	100%	0.22
4	**-0.18**	100%	0.06	**1.63**	87%	3.64	**0.02**	30%	0.48	**4.06**	100%	0.21
5	**-0.12**	100%	0.03	**6.01**	97%	2.24	**0.65**	80%	0.88	**4.01**	100%	0.26
6	**-0.20**	100%	0.05	**2.46**	97%	1.46	**0.91**	93%	0.82	**3.82**	100%	0.34
7	**-0.17**	100%	0.03	**3.21**	100%	1.20	**0.96**	93%	0.92	**3.89**	100%	0.27
8	**-0.17**	100%	0.03	**4.09**	100%	1.38	**1.18**	90%	0.51	**3.84**	100%	0.24
9	**-0.06**	100%	0.02	**1.10**	93%	0.34	**0.28**	40%	0.27	**1.72**	100%	0.02
10	**-0.16**	100%	0.03	**2.28**	100%	0.88	**0.78**	93%	0.36	**4.11**	100%	0.23
11	**-0.20**	100%	0.03	**2.60**	100%	0.89	**0.75**	87%	0.71	**3.90**	100%	0.21
12	**-0.16**	100%	0.04	**4.18**	100%	1.09	**1.44**	97%	0.69	**3.87**	100%	0.25
13	**-0.16**	100%	0.04	**4.85**	100%	1.66	**1.44**	90%	1.51	**4.02**	100%	0.26

Our results confirm that firm size decreases the risk of mortality [36, 37]. Moreover, market concentration, measured with the Gini index, increases mortality for all firms, large and small alike. *S* firms have a higher risk of mortality than *L* firms over the whole simulation time. Moreover, we observe that a firm’s distance to the market center monotonically increases its mortality risk.

#### Outcome 3

Mortality risk decreases with firm size, and increases with market concentration and with the distance to the market center.

The simulation results demonstrated that very few *L* firms succeeded in taking over the center. The majority, mostly narrow-niche (and small-sized) *L* firms, typically stayed at the market fringes, and had a short live. However, the few *L* firm survivors had become the strongest and largest firms in the market. The strongest *S* firms are those that have benefited from scale economies (reaching a relatively large size of 50–100 units). However, they still remained located at the market periphery. To investigate the differential impact of firm size on firm type as market concentration increases, we estimated models with three-way interaction terms of *Type* × *Gini* × *Size*. The minimum and maximum coefficient values are presented in Table 3. We found all coefficients to be negative and significant at *α* = 0.05. This implies that *S* firms’ mortality hazard would, ceteris paribus, increase with concentration, but this extra hazard is offset by their size gain effect.

**Table 3:** Interaction effects.

Type x Gini x Size coefficient		
Scenario	Min. value	Max. value
1	-0.696	-0.163
2	-2.265	-0.744
3	-0.654	-0.179
4	-0.694	-0.201
5	-0.439	-0.141
6	-0.851	-0.179
7	-0.707	-0.103
8	-0.705	-0.221
9	-0.186	-0.094
10	-0.461	-0.185
11	-0.806	-0.322
12	-0.630	-0.160
13	-1.192	-0.324

**Fig 11:**
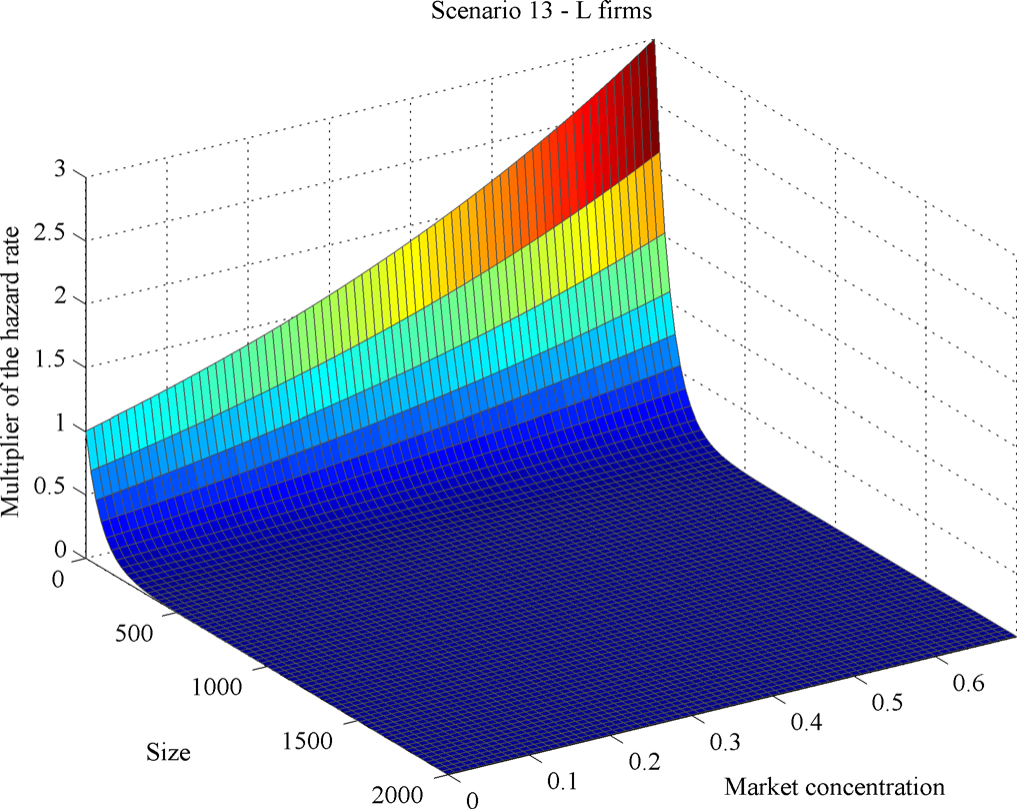
Dependency of mortality effects on market concentration and size for scenario 13: *L* firms.

**Fig 12:**
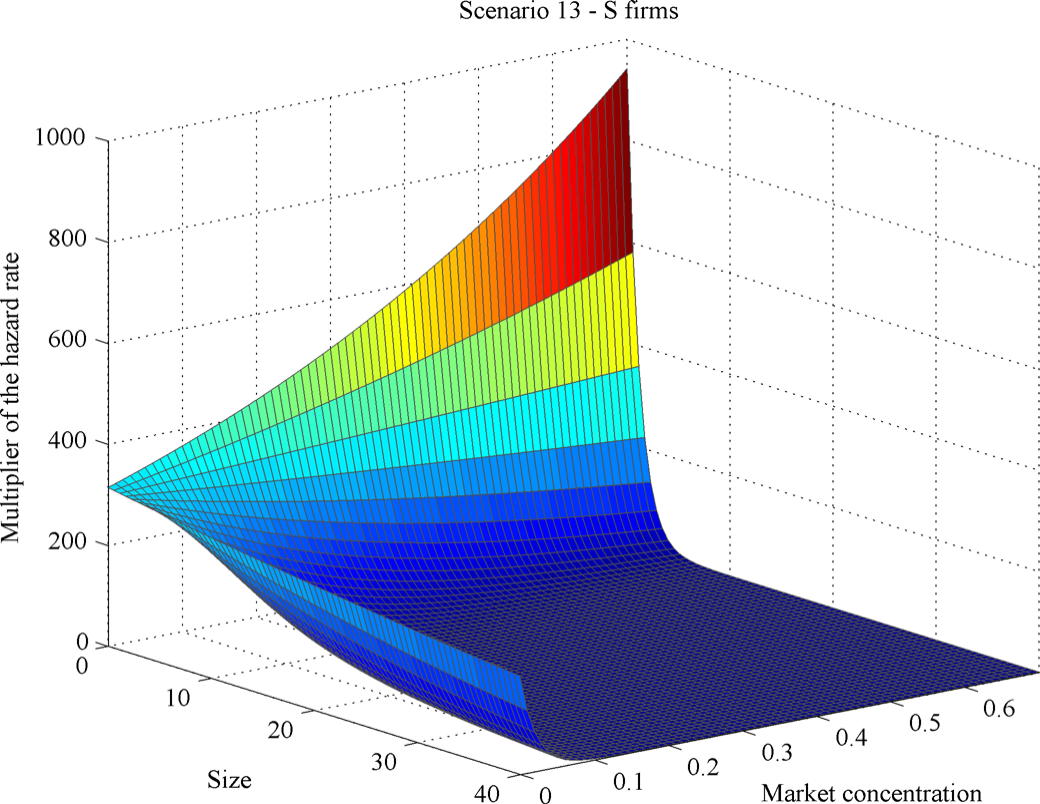
Dependency of mortality effects on market concentration and size for scenario 13: *S* firms.

**Fig 13:**
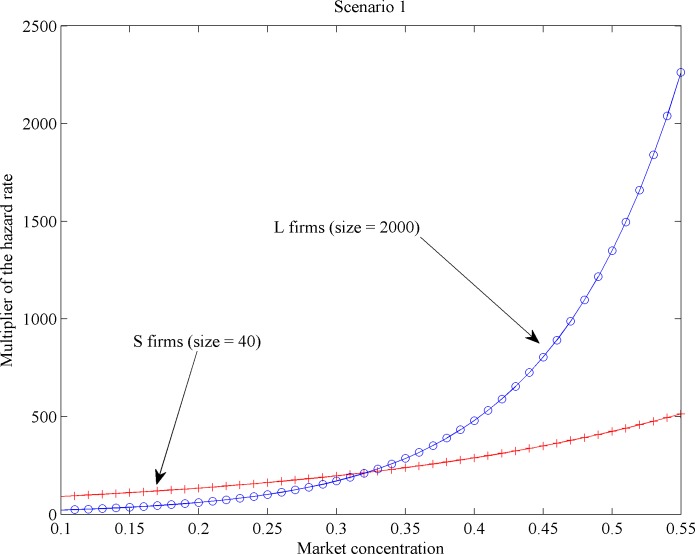
Mortality effects with increasing concentration per firm type: Scenario 1.

**Fig 14:**
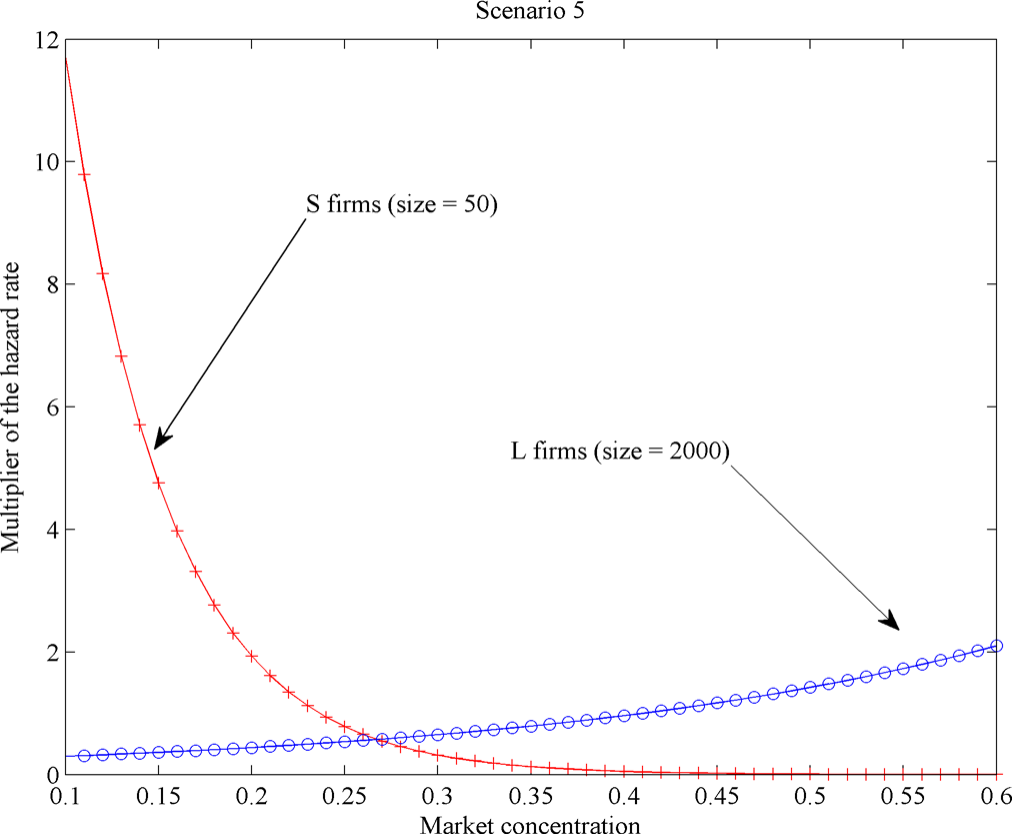
Mortality effects with increasing concentration per firm type: Scenario 5.

Additionally, we computed the so-called hazard rate multipliers per scenario (see [34]). Generically speaking, hazard rate multipliers quantify the mortality effect of a given variable normalized by the lowest mortality value produced by that variable. Here, hazard rate multipliers account for the effect of market concentration on the mortality rate per firm type across different firm sizes, as illustrated in Figs 11–14. We found across all scenarios that large-sized *S* firms had lower mortality risks than small-sized ones as market concentration goes up. The direction of the concentration effect on mortality hazard effect was also size specific: whereas the smallest *S* firms experienced higher mortality hazards with rising concentration, the largest ones faced a decrease. For *L* firms, the impact of firm size on mortality varied over scenarios. But the *L* firm multiplier did not decline with firm size under any scenario (Figs 11 and 12). These findings allow for different possibilities for the case of increasing market concentration: either the mortality hazard increases slower for the largest *S* firms than for *L* firms (Fig 13), or the mortality hazard decreases for the largest *S* firms whilst increasing for all *L* firms irrespective of their size (Fig 14).

#### Outcome 4

As market concentration increases, either (i) large *S* firms’ mortality hazard decreases whilst that of *L* firms’ does increase or (ii) *S* firms’ mortality hazard increases at a slower pace than that of *L* firms; (iii) the smallest *S* firms’ mortality hazard always increases with concentration.

So dual market structure may also develop when increasing concentration raises *S* firms’ hazard less than that of *L* firms. This outcome indicates the existence of an endogenous *minimal scale of production* as an emergent property in our simulation model: the very small players disappear. Small niche players falling below this minimum scale cannot benefit from the space release induced by increasing concentration. Thus the dual structure is built up of large and small (but not extremely small) firms.

Additionally, outcome 4 reveals that size effects operate differently on *L* vis-à-vis *S* firms. For *L* firms, increasing size weakens the concentration-related mortality effect; however, the effect always stays positive. For *S* firms, concentration might either increase or decrease mortality. The broad range of size-related simulation outcomes we have studied reveals that two types of partitioning, a weak and a strong version, can emerge. As market concentration rises, either *L* firms and *S* firms experience increasing and decreasing mortality (strong partitioning), respectively, or both experience increasing mortality with *S* firms’ chances deteriorating at a slower pace (weak partitioning). This finding resembles to the strong and weak patterns of partitioning observed in the dual-market study of [34] on Dutch auditing firms.

### Regression Results

As explained above, our OLS regression analysis takes the simulation model’s parameters as independent variables out of 3,240 observations. In 17 observations, the market has become extinct long before the end of the simulation’s time horizon. Our four dependent variables were, again, average market concentration (*Gini*), average *L* and *S* firm density over the last 500 time periods, and space release. The last effect is proxied with *L* firm space contraction (*Lcontr*), which is the difference between maximum and average space occupied by *L* firms in the last 500 time periods.

Our estimates are presented in Table 4. The observed minor non-normality of the residuals is not relevant as we have a large number of observations. In order to deal with heteroskedasticity, we ran our regression with robust standard errors whilst iteratively computing weighted least squares. The results were quite similar. Here, we only report the OLS estimates. Market concentration was found to be negatively correlated with the small sunk cost parameter *Q*_*S*_ and product dissimilarity *γ*. This is in line with expectations, since increasing *S* firm scale advantages and distance-related effects both lower the scale advantage of *L* firms.

**Table 4:** Regression results.

Independent variables		Concentration (Gini) index	*L* density	*S* density	*L* space contraction
*Q*_*S*_	Small sunk cost parameter	-.0175831*	-.1431061*	2.774796*	1.363785*
		(.0002214)	(.0045447)	(.0448818)	(.0217619)
*γ*	Product dissimilarity	-.0025169*	.0045806	.5720272*	.1815882*
		(.0001565)	(.0031975)	(.0340542)	(.0149023)
*φ*	Markup factor	.1163989*	-1.44986*	76.54684*	9.791984*
		(.0155546)	(.3160434)	(3.394657)	(1.464913)
*ExpCoef*	Expansion coefficient	3.238009*	8.51728*	-263.9791*	-121.3129*
		(.1568174)	(3.169922)	(34.50324)	(14.7435)
*E*	Endowment	.00345*	1.250871*	.0937271	.6340934*
		(.0002614)	(.0061466)	(.0563541)	(.0244667)
*X*	Entry rate	.0007879*	5.255838*	7.353022*	3.898299*
		(.0025293)	(.0516949)	(.5532305)	(.2395199)
Intercept		.8440625*	-9.539741*	-106.2712*	-39.89156*
		(.0233112)	(.4662876)	(5.094417)	(2.274647)
Number of observations		3223	3223	3223	3223
F(6, 3126)		1125.17	8786.44	801.88	784.76
R^2^		0.6845	0.9545	0.5640	0.6199
Root MSE		0.07178	1.4674	15.711	6.8014

Robust standard errors in parenthesis

* *p* < 0.05.

As expected, *L*-firm space contraction (*Lcontr*) is larger when *Qs* is high, since increasing *S* firm scale decreases the competitive advantage of *L* firms. Our interpretation is that *L* firm space contraction is partially due to their direct competition with *S* firms. This also suggests that *L*-firm’s space release in the peripheries is not necessarily triggered by increasing market concentration, as quite a few earlier studies observed in industries with a generalist-specialist dual structure [2, 18]. Here, our generic *L*-*S* firm model identifies broader conditions under which dual structures can occur. We found a positive correlation between *L* firms’ endowment (*E*) and the contraction of space occupied by these *L* firms. Higher endowment provides larger endurance for *L* firms, offering protection against the negative effects of market center crowding, thus allowing them to stand longer in the crowded market center. A higher capacity to endure competition at the center intensifies competitive overlap and triggers subsequent demand release in the peripheral market areas. A recent empirical study of Italian television broadcasting [38] illustrates that intensification of competitive overlap at the market center alone does support space release at the peripheries, even with concentration remaining invariant. In line with our simulation results, this empirical example supports the finding that large *L* firm endowment may even help small firms to gain and maintain a foothold at the market fringes.

Moreover, we found that as the expansion coefficient (*ExpCoef*) increases, *L* firms grow faster, leading to lower *S* firm density. Additionally, our results indicate that *L* firms’ larger expansion capabilities come together with their smaller space loss; their higher expansion probability is not only associated with their ability to move toward the market center, but also with their larger niche position takeover and, consequently, with having less space left for *S* firms to grow.

#### Outcome 5

*L* firms’ higher expansion probability and higher endowment allows them to move toward, and endure competition in, the market; a higher *L* firm expansion probability decreases *S* firms’ space release at the peripheries, while higher *L* firm endowment favors this space release.

Outcome 5 suggests that enhancing the adaptive capacity of central market players to compete has two opposing effects, depending on whether the enhanced capacity comes from resilience (captured by endowment) or from flexibility (captured by expansion probability). On the one hand, increasing competitive resilience may increase *L* firms’ tolerance to near-center overlap, consequently lessening their interest in occupying positions at the market peripheries. On the other hand, adaptation through higher expansion abilities allows *L* firms to move easier across space, so impacting negatively on *S* firms’ space covering. Our results indicate that assigning different levels of adaptive capabilities to firms through varying endowment and expansion abilities can extend the range of possible dual market outcomes. On the basis of the simulation findings, we hypothesize that adaptive capability differences across firms can weaken or reinforce partitioning outcomes in a broad range of industries.

## Conclusions

Our simulation results demonstrate that the set of large, typically generalist, firms to a large extent coincides with the set of large sunk cost firms whenever the microeconomic conditions adopted by our model are combined with a market center-periphery distinction. Several of our simulation settings yielded dual market outcomes that are similar to those predicted by the known dual market approaches surveyed in the introduction. This is, very likely, a consequence of the resemblance of our model assumptions to fundamental premises of these other theories. For example, our firms are characterized by a low/high sunk cost distinction (just as at [1]) interacting in a market with a unimodal demand landscape (just as at [2, 17, 38]). But the simulations have also revealed aspects of dual market formation, which, according to our knowledge, have not yet been addressed by other theories.

The results corroborating extant knowledge are as follows. Market concentration, measured with the Gini index, increases mortality for all firms, large and small alike (Outcome 3). This is line with IO’s argument as to the entry barrier and market power effect, as well as with main effect results from empirical market partitioning studies (like [34] on the dual market structure of the Dutch auditing industry). We observed space release at the market peripheries with scale-based competition (Outcome 2) and with subsequent large firm shake-out at the market center. This shows a clear analogy to documented cases of dual structure formation with center and peripheral market players [2] (Outcome 1). Even with a modest space release in place, small *S* firms experience decreasing mortality rates with rising market concentration, while large *L* firms’ mortality hazard increases in the meantime (Outcome 4). Distance to the center is a determinant of the mortality hazard (Outcome 3). Also in line with extant knowledge, we found that a noticeable level of firm heterogeneity–in our case, measured in terms of sunk cost magnitudes–is necessary to generate a center-periphery market partitioning (cf. [22]).

The next findings reflect new insights that add to the extant literature on dual market formation in particular, and to theories of market dynamics in general. The first concerns the role of price mechanisms in market partitioning processes. From the very beginning [2], resource-partitioning theory has focused on markets where price was not the central aspect for customers (like in case of newspapers and microbreweries, [15, 17]) or where price effects are suppressed by other aspects such as reputation or the bundling of the offerings (e.g., in case of auditing services, [34]). We have demonstrated that a dual market structure with center-periphery partitioning can well occur when agents are sensitive to price.

Second, the effect of concentration on *L/S* firm types is moderated by a size effect. We found that that there is an emergent (endogenous) minimum scale of production that allows *S* firms to withstand the negative effects of market concentration, as well as their larger distance to the market center (Outcome 4). So, size effects can establish a concentration-dependent mortality threshold. Thus, although the smallest *S* firms might though avoid head-on competition with market-center players, they still may succumb to other, somewhat larger, *S* firms.

Third, we found a temporally non-monotonic space (i.e., demand) release effect (Outcome 2). *L* firms may first move out from the center in order to lessen competition, so occupying more rather than less (semi-)periphery positions. This happens because large sunk cost *L* firms might not yet operate close to their full scale potentials; their ‘efficiency slack’ can be used up to counterbalance scope diseconomies, and to expand, in the early consolidation phase. Occasionally, *L* firms may even take over more space than had been released by them. Later, when shake-out frees up additional affluent center demand that survivor *L* firms can efficiently capture, the relative advantages of aiming at scarcer periphery demand become weaker, while *L* firms’ scope diseconomies at the peripheries remain the same. In this mature phase, we found the space occupied by *L* firms contracting. The bottom line is that scale-based competition at the market center, alone, might not be enough to generate space release. Moreover, *L* firms may operate below their optimal scale when market concentration just begins to rise, so that space release might just partially emerge. Then, space gains, if they occur, come at the expense of *L* firms failing because of the pressure of those *S* firms that could reach a certain size. Again, size effects per firm type operate in such a subtle way that while *L* firms do not get any benefit from increasing market concentration, some larger *S* firms’ mortality hazard may decline.

Fourth, our model introduced firm-level adaptive capabilities through endowment or increased expansion chances, demonstrating that the relative magnitude of these two capabilities of *L* firms have effects on their space release at the market peripheries (Outcome 5). Previous research [5] had claimed that the nature and degree of (scale and) scope economies are key in understanding the precise equilibrium market structure outcomes. Here, we add that the interplay between market-level selection forces and firm-level adaptive capabilities may lead to a large number of new, and different, dual market formation scenarios.

## Supporting Information

S1 File Supporting information file. In the S1 File we present further details about the computer simulation model, including parameter values.
(PDF)
