# Supplementary material for: Micro-Level Adaptation, Macro-Level Selection, and the Dynamics of Market Partitioning
Source: PLoS One. 2015 Dec 14;10(12):e0144574. doi: 10.1371/journal.pone.0144574 (PMC4687648; doi:10.1371/journal.pone.0144574)
Supplement: S1 File — In the S1 File we present further details about the computer simulation model, including parameter values. (PDF) [file pone.0144574.s001.pdf]

S1 File

Supporting Information

Extended conceptual model description

The resource space and location specification at entry

The resource space corresponds to a unimodal distribution of consumers along a set of taste preferences of size *N*. Each taste preference has a number b_k of consumers, $k = 1,2, \ldots, N$. The resource space is set according to a beta distribution with parameters $\alpha^* = \beta^* = \eta$. In order to get a unimodal representation we use $\eta = 3$ and $N = 100$. Firms enter the market at a constant rate *x* per time period provided that the space is not completely occupied; otherwise (i.e., when the market is fully saturated) there is no entry. All the simulation runs are performed using a total demand of $\sum_k b_k = 5500$ consumers. Firms that enter the market pick up their initial location according to the probability distribution of non-served consumers, $\boldsymbol{\rho_t}$, which is given by:

$$\rho_{k,t} = \frac{(1 - CBP_{k,t-1})b_k}{\sum_{i=1}^{N}(1 - CBP_{i,t-1})b_i}, \quad \forall k = 1,2,\ldots,N\,, \tag{A1}$$

where $CBP_{k,t\text{-}1}$ represents the active consumer base percentage at position *k* at time *t-1*. Since at the beginning of the simulation (t = 0) there is no active consumer base, then $\rho_{k,0} = b_k/\sum b_i$.

Firm’s cost structure

Firms have a two-piece cost function. One piece relates to the production costs $C^i_{PROD,t}$, and the other one accounts for the niche-width costs, $C^i_{NW,t}$,:

$$C^i_t(Q) = C^i_{PROD,t}(Q) + C^i_{NW,t}\,. \tag{A2}$$

Production levels *Q* are quantified through a Cobb-Douglas function:

$$Q_{i,t} = F_i^{\alpha} V_{i,t}^{\ \beta}\,. \tag{A3}$$

Coefficients α and β correspond to production volume elasticities with respect to production factors *F* and *V* (that is, $\alpha = (\partial Q/\partial F)(F/Q)$ and $\beta = (\partial Q/\partial V)(F/Q)$). Firms derive their production costs, $C^i_{PROD,t}$, from a long-run average cost curve, *LRAC*, of the

whole industry. The *LRAC* curve is the envelope of the most efficient production possibilities in the industry. We make $\alpha + \beta > 1$ in order to reflect a downward-sloping *LRAC* and positive scale economies. Production costs for the firm are calculated according to the usage of production factors amounts *F* and *V* that the firm needs to produce quantity *Q*. That is, assuming that production factor prices are W_F and W_V, respectively, the *LRAC* curve is calculated by solving the following optimization problem:

$$\begin{aligned} \min \quad & W_F F_i + W_V V_i \\ s.t. \quad & Q_{i,t} = F_i^{\alpha} V_{i,t}^{\ \beta} \end{aligned} . \tag{A4}$$

Parameters W_V, W_F, α and β are set in order to obtain a (normalized) unit cost of 1 when $Q = \sum_i b_i$ ($W_V = 4.15$, $W_F = 2W_V$, $\alpha = \beta = 0.7$). The production cost of every firm i, $C^i_{PROD,t}$, is also computed through Equation (A4), but then assuming that the firm has a fixed usage of factor *F*, independent from production levels (that is, $W_F F_i$ represents firm's fixed costs). Firms may have two different alternatives to define the usage of factor *F*: a large (*L*) and a small one (*S*). These two options define the two different firm types in the model. Each one of the two possible values of *F* is set according to the quantity *Q* at which the firm's cost curve and the *LRAC* intersect. The model assumes that the large fixed cost indicator, Q_L, is set at least at half of the total market demand, $Q_L \geq \sum_i b_i/2$, while the small sunk cost value, Q_S, varies from quantities as low as 5, $Q_S \geq 5$. The baseline model uses $Q_L = \sum_i b_i/2$ and $Q_S = 10$. However, model's behavior is also inspected under different "scale distances" (Q_L - Q_S). An entrant has equal probability to select either firm type.

Niche-width costs represent the negative effect of covering a market with a large scope of consumer preferences. That is, a firm finds it more expensive to cover a highly diverse consumer-preference market than a homogenous one. Niche-width costs are defined as a function of the upper and lower limits of firm *i*'s niche, and a proportionality constant *NWC*:

$$C^i_{NW,t} = NWC \left\| w^u_{i,t} - w^l_{i,t} \right\| , \tag{A5}$$

where $\|.\|$ represents the (Euclidean) distance between the two niche limits, and $w^l_{i,t}$ and $w^u_{i,t}$ represent the firm's lower and upper niche limits. Firm *i*'s niche center is then defined as $nc^i_t = \left\| w^u_{i,t} - w^l_{i,t} \right\| / 2 + w^l_{i,t}$.

Consumer behavior

Each consumer buys only once every time period. Assuming that the selected firm has still enough produced units to cover demand, and that $S_{k,t}$ represents the set of firms that have an offer at position *k*, a consumer evaluates the offerings at his or her location *k*. The consumer buys from the firm that offers the lowest compound cost (price plus product dissimilarity) from the set of options $\{U^i_{k,t}\}$ (i.e., considering all the *i*-th firms that belong to the set $S_{k,t}$):

$$U^*_{k,t} = \min_{i \in S_{k,t}} \{U^i_{k,t}\} = \min_{i \in S_{k,t}} \left\{ P^i_t + \gamma \frac{\| nc^i_t - k \|}{(N-1)} \right\}, \qquad \text{(A6)}$$

where P^i_t is the firm *i*'s price at time *t*, and γ is a constant that quantifies the effect of distant offerings in the space from the firm's niche center (product dissimilarity). The distance-related effect is normalized over the maximum possible Euclidean distance in the model, N - 1. In case that the selected firm does not have enough produced units to satisfy a consumer, the consumer decides to buy from the second cheapest alternative, and so on. To avoid any synchronization artifact, order positions for the buying process are randomly permuted every time period.

The reader might ask why apparently the distance effect is counted twice: firms have a negative scope effect through the niche-width cost, but also are penalized through the product dissimilarity effect. The two settings $\{\gamma > 0, NWC = 0\}$ and $\{\gamma > 0, NWC > 0\}$ produce rather similar results: Both revealed in the long run that *L* firms basically take over the center while *S* firms locate at periphery. This means that the inclusion of *NWC* does not influence the scale-based selection process of the model. However, only the setting $\{\gamma > 0, NWC > 0\}$ revealed a sharp niche-width difference between firms located at the periphery and those located at the center. Therefore, we adopt such a setting $\{\gamma > 0, NWC > 0\}$, since it resembles more precisely what resource-partitioning theory argues: Location in the space is related to the degree of niche-width differentiation (generalism / specialism).

For the sake of simplicity, we do not use demand functions, but define a limit price value for firm operations. The maximum price a consumer is willing to pay corresponds to a opportunity cost of the smallest efficient firm in the industry $P_{max} = (1+\varphi)LRAC\big|_{Q=1}$. This implies that the only reason a consumer would buy from a larger

firm is that such a firm is more cost-efficient than the smallest possible firm in the industry (i.e., the firm's "scale" Q is located rightward along the *LRAC* curve). That said, consumers are allowed to bear a maximum cost U_o, so that $U^i_{k,t} \leq U_o = P_{max}$. The amount U_o defines a cost-related participation constraint for consumers. In general, if the consumer chooses to buy from a firm i^* whose niche center does not coincide with the consumer's preference k, the price $P^{i^*}_t$ has to comply with

$$P_t^{i*} \leq P_{\max} - \gamma \frac{\left\| nc_t^i - k \right\|}{(N-1)}. \tag{A7}$$

In order to reflect scale advantages, firms use a markup price over average costs (i.e., $(1+\varphi)C(Q)/Q$), provided that the markup price complies with Equation (A7). Coefficient φ may range between 0 and 1.

We calibrated coefficients *NWC* and γ following these steps: (i) Since we assume that L firms are more efficient than S firms, we also assume that in absence of distance-related dissimilarity ($\gamma = 0$), a fully-expanded L firm should be able to outcompete any S firm. Experimentation with the model reveals that, to comply with that, the maximum value that the maximum vale the *NWC* coefficient can take is 195; (ii) with $NWC = 195$, the value range of coefficient γ was specified by assuming that the *fundamental niche* of an L firm (i.e., the space the firm would occupy in absence of any competition) should oscillate between half and two-thirds of the total resource space (that is, $\gamma \in [50, 70]$).

Entry price setup

Firms enter at one single position in the space. As seen in Equation (A1), we assume that firms search for a competitor-free foothold to enter the market. Firms pay attention to residual demands – the amount of non-served consumers – at different points in the resource space. From Equation (A1), we observe that the probability they step in a given location increases with the size of the residual demand spot. If the selected position for entry is position k, the firm considers a potential production quantity $Q = (1 - CBP_k)b_k$, which corresponds to the residual demand. The firm fixes a unit price that corresponds to $\min\{P_{max}, (1 + \varphi)C(Q)/Q\}$.

L firms may need some time to grow and reach an operation point that allows them to sustain positive profits. S firms are able to make profits at the time they enter

the market. *L* firms have negative profits until they reach a minimal operational point. Thus, we assume that *L* firms have an initial *endowment*. Endowment is implemented as a number *E* of periods for which a firm can survive in case of no sales (that is, covering fixed costs for *E* time periods). For the baseline model, $E = 12$, but other values are explored in order to investigate implications on the model's results.

Firm expansion

There are two possible ways for a firm to expand: vertical and horizontal. The firm uses an adaptive "rule of thumb", based on the latest information of the market, to assess if expansion is worth the investment. Being the expansion either vertical or horizontal, the firm first defines a *target quantity* in terms of the latest observed prices and costs. Based on such a quantity, the firm computes incremental profits and decides whether or not expansion is worth the investment.

(i) **Vertical expansion** refers to a niche production quantity adjustment. At time *t*, firm *i* makes production adjustments for the next round and targets the residual demand $\Delta Q_{v,t+1}$ – the amount of non-served consumers – in their current niche $H_{i,t}$, so that $\Delta Q_{v,t+1} = \sum_{k\in Hi,t}(1-CBP_{k,t})b_k$. Then, if Q_t represents firm *i*'s latest sold quantity, the firm evaluates whether incremental revenues surpass incremental costs. If that is the case, the firm expands. Incremental costs are computed as $C^i(Q_t + \Delta Q_{v,t+1}) - C^i(Q_t)$. Incremental revenues are computed as $P^{i*}(Q_t+\Delta Q_{v,t+1}) - P^i_t Q_t$, where P^{i*} is calculated as follows:

$$P^{i*} = \min\left\{ P_{\max} - \gamma \frac{\left\| nc_t^i - w_{i,t}^l \right\|}{(N-1)}, (1+\varphi)C(Q_t + \Delta Q_{v,t+1}) \right\}. \quad \text{(A8)}$$

That is, firms use a markup price as long as it does not exceeds the maximum allowed price according to the width of the firm's current niche (see Equation (A8)).

(ii) **Horizontal expansion** refers to niche expansion. It also establishes a target quantity $\Delta Q^u_{h,t+1}$ and $\Delta Q^l_{h,t+1}$ on either side of the current firm's niche (upper and lower limits), respectively. The firm decides to expand toward the most attractive direction – that is, to the position where incremental profits are larger – in a similar fashion shown for vertical expansion. Firms do not always expand, so that expansion is controlled by an expansion probability, *ExpCoef*, at every time period. Values for the coefficient

ExpCoef were jointly selected along with the time-horizon span over which we expected to see a convergence of market concentration and firm density. Since we run the model for 2000 time periods, our criteria is that an *L* firm should have enough time to fully expand to its fundamental niche, even if it enter the market at a mature state (> 1000 time periods). Values were chosen between 0.03 and 0.05. This coefficient might be also related to the firm's degree of inertia. Along with the results reported here, it is worth mentioning that our simulation trials confirmed a location-related selection process – *L* firms taking over the center and *S* firms dominating the periphery – even in absence of any inertia effects (i.e., *ExpCoef* = 1).

In the case a firm decides to expand, it evaluates in which direction to go. The quantities $\Delta Q^{u}_{h,t+1}$ and $\Delta Q^{l}_{h,t+1}$ are set according to the same set of rules. Let us assume that firm *i* attempts expansion to the position adjacent to its upper niche limit, *z*. If the targeted position *z* is empty, then $\Delta Q^{u}_{h,t+1} = b_z$. If some firms are already at position *z*, then the expanding firm *i* estimates $\Delta Q^{u}_{h,t+1}$ by taking into account the rival's costs $U^{j}_{z,t}$, and rivals' latest sold quantities, at position *z*, which firm *i* assumes to be the best estimates of their next time-period quantities. Then, the offered costs are compared and ranked, and the quantity $\Delta Q^{u}_{h,t+1}$ is subsequently extracted according to the relative rank the firm gets in comparison with the rivals' costs. An example of how this is carried out follows next. Let us assume that the location of interest has a total demand of ten consumers, and the compound costs at that position from two different firms are $U(A) = 10$ and $U(B) = 15$ with captured demands $Q(A) = 7$ and $Q(B) = 3$. If firm *C* attempts to enter that position, and assuming that $U(C) = 12$, the ascendant cost ranking will place firms in the following order: *A*, *C*, and *B*. Firm *C* estimates that *A* will keep its last demand in the next round (i.e., $Q(A) = 7$), since *A* still has the cheapest offer. But now, given that *C* has a better offer than *B*, *C* will steal B's demand and estimate that in the next round $Q'(C) = 3$ and $Q'(B) = 0$. Once the target quantity has been established for *z*, the firm calculates its potential incremental profits by taking into account (i) the resulting total costs with the added target, (ii) the resulting price, including the new distance compensation $\gamma \left\| nc^{i}_{(Hi,t)+z} - z \right\| / (N-1)$, where $nc^{i}_{Hi,t+z}$ is the new niche center that would result if *z* is included in firm *i*'s niche, $H_{i,t}$, and (ii) the firm's latest price and costs. The firm then computes incremental profits and performs the same calculation when expansion is attempted to the cell adjacent to the lower limit. After comparing the

two incremental profits, the firm decides to expand toward the position that reveals the highest value, if any.

Analysis

We study the effect on hazard rates according to different representative scenarios, which are set according to variations in small sunk cost investment (Q_S), product dissimilarity coefficient (γ), endowment (E), entry rate (X), markup value (φ) and expansion probability (*ExpCoef*). The scenarios correspond to a model with mid-range parameters (scenario 1), variations in the small sunk cost investment (scenarios 2, 3 and 4), markup (scenarios 5 and 6), product dissimilarity (scenarios 7 and 8), endowment (models 9 and 10), probability of expansion (scenarios 11 and 12) and entry rate (scenario 13). Every scenario is run for 2000 time periods. Within every scenario, we also studied the behavior of main time-evolving variables of interest (i.e., market concentration, per-type density and per-type total covered space). Every scenario is run 30 times in order to guarantee the normality assumption of confidence intervals of such variables. We find survival analysis estimators for each realization of the 30 x 13 = 390 simulations. See S1 Table for details.

Table A. Parameter values for model dynamics analysis

**Parameter**	**Definition**	**1**	**2**	**3**	**4**	**5**	**6**	**7**	**8**	**9**	**10**	**11**	**12**	**13**
Q_{SS}	Small sunk cost	10	**5**	**15**	**20**	10	10	10	10	10	10	10	10	10
φ	Markup	0.2	0.2	0.2	0.2	**0.1**	**0.3**	0.2	0.2	0.2	0.2	0.2	0.2	0.2
γ	Product dissimilarity	60	60	60	60	60	60	**50**	**70**	60	60	60	60	60
E	Endowment	12	12	12	12	12	12	12	12	**6**	**18**	12	12	12
ExpCoef	Expansion probability	0.04	0.04	0.04	0.04	0.04	0.04	0.04	0.04	0.04	0.04	**0.03**	**0.05**	0.04
x	Entry rate	3	3	3	3	3	3	3	3	3	3	3	3	**2**

The second analysis consists of exploring effects in four specified outcomes: market concentration, L firm density, S firm density, and L firm space contraction. Space contraction is evaluated as the difference between the peak space occupation and the average occupation at the final time steps of the simulation. Effects are explored with respect to variations in the simulation key parameters: small sunk cost (Q_S), product dissimilarity (γ), markup (φ), endowment (E), Expansion probability (*ExpCoef*),

and entry rate (*x*). Using the selected values presented in S2 Table, we get 4 x 3^4 x 2 = 648 combinations for the above-mentioned parameters, respectively. We build an OLS regression model using the below-mentioned parameters as independent variables, using the earlier mentioned four outcomes of interest

Table B. Parameter values for OLS analysis

Parameter	Definition	Selected values
Q_{SS}	Small sunk cost parameter	5, 10, 15, 20
γ	Product dissimilarity	50, 60, 70
φ	Markup factor	0.1, 0.2, 0.3
ExpCoef	Expansion coefficient	0.03, 0.04, 0.05
E	Endowment	6, 12, 18
x	Entry rate	2,3

Every parameter combination was replicated *m* = 5 times. Thus, the total number of observations in this analysis is 648 x 5 = 3240. To guarantee more precise effects on convergence conditions, we run each simulation for 5000 time periods, and take averages on the last 500 periods on the key outcome variables (i.e., the last 10% fraction of the total simulation time).
